# Extrapair paternity in two populations of the socially monogamous Thorn‐tailed Rayadito *Aphrastura spinicauda* (Passeriformes: Furnariidae)

**DOI:** 10.1002/ece3.6850

**Published:** 2020-09-29

**Authors:** Esteban Botero‐Delgadillo, Verónica Quirici, Yanina Poblete, Silvina Ippi, Bart Kempenaers, Rodrigo A. Vásquez

**Affiliations:** ^1^ Department of Behavioural Ecology and Evolutionary Genetics Max Plank Institute for Ornithology Seewiesen Germany; ^2^ Instituto de Ecología y Biodiversidad Departamento de Ciencias Ecológicas, Facultad de Ciencias Universidad de Chile Santiago Chile; ^3^ SELVA: Research for conservation in the Neotropics Bogotá Colombia; ^4^ Departamento de Ecología y Biodiversidad, Facultad de Ciencias de la Vida Universidad Andrés Bello Santiago Chile; ^5^ Centro de investigación para la sustentabilida, Universidad Andrés Bello Santiago Chile; ^6^ Instituto de Ciencias Naturales Universidad de las Américas Santiago Chile; ^7^ Departamento de Zoología CRUB Universidad Nacional del Comahue – CONICET Bariloche Argentina

**Keywords:** breeding density, Furnarioidea, intraspecific variation, mating system, reproductive strategy

## Abstract

Studies on extrapair paternity (EPP) are key to understanding the ecological and evolutionary drivers of variation in avian mating strategies, but information is currently lacking for most tropical and subtropical taxa. We describe the occurrence of EPP in two populations of a South American socially monogamous bird, the Thorn‐tailed Rayadito, based on data from 266 broods and 895 offspring that were sampled during six breeding seasons in north‐central and southern Chile. In the northern population, 21% of the broods contained at least one extrapair young and 14% of all offspring were sired by an extrapair male, while in the southern population, we detected extrapair offspring (EPO) in 14% of the broods, and 6% of all offspring were EPO. Variation in the frequency of EPP could stem from population differences in the duration of the breeding season or the density of breeding individuals. Other factors such as differences in breeding synchrony and variation in food availability need to be evaluated. More reports on EPP rates are necessary to determine the patterns of taxonomic and geographic variation in mating strategies in Neotropical birds, and to better understand the differences in ecological dynamics between northern and southern hemisphere populations.

## Graphical abstract

Information on extrapair paternity (EPP) is currently lacking for most tropical and sub‐tropical birds. Based on data from 266 broods and 895 offspring that were sampled during six breeding seasons in north‐central and southern Chile, we describe the occurrence of EPP in two populations of a South American socially monogamous bird, the Thorn‐tailed Rayadito. We found EPP rates higher than in other tracheophone Suboscines, and observed intraspecific variation that could be related to differences in the duration of the breeding season or the density of breeding individuals.

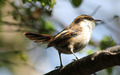

## 1 INTRODUCTION

The study of genetic mating systems is critical to understand how selection has driven variation in bird breeding behavior. Social monogamy with biparental care is the most widespread avian mating system (Lack, 1968; Ligon, 1999), observed in 81% of all avian species (Cockburn, 2006). However, hundreds of studies have revealed that genetic polyandry is prevalent in species exhibiting this social mating system (reviewed in Brouwer & Griffith, 2019; Griffith et al., 2002; Spottiswoode & Møller, 2004). Among 342 socially monogamous species for which parentage has been genetically determined, extrapair offspring (EPO) have been detected in 75% of the cases (Brouwer & Griffith, 2019).

In a recent review of extrapair paternity (EPP) in birds, Brouwer and Griffith (2019) showed that ample data exist on EPP rates of some temperate taxa (e.g., Great Tits *Parus major* or Blue Tits *Cyanistes caeruleus*), but that information on EPP is currently lacking for ~96% of all described avian species. Moreover, while some bird families—mostly those from temperate regions—are over‐represented in those studies, 56% of all recognized families have yet to be investigated. Brouwer and Griffith (2019) also highlighted that only 17% of all published studies on EPP were on species from South America or Africa. As this bias may hinder a complete understanding of the prevalence of EPP and the ecological and evolutionary drivers of variation in avian genetic mating systems, more studies focused on tropical and subtropical bird taxa are needed (Brouwer & Griffith, 2019).

**Figure 1:**
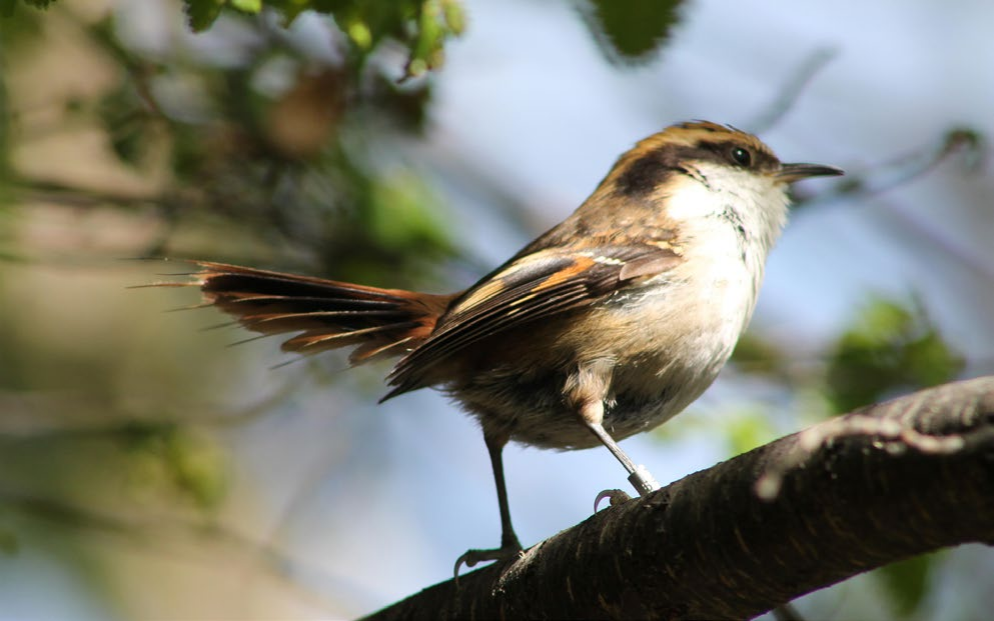
Breeding adult of Thorn‐tailed Rayadito in Navarino Island. Female and male rayaditos do not exhibit obvious sexual dimorphism. Photo by Yanina Poblete

We here report on the occurrence of EPP in two populations of the Thorn‐tailed Rayadito (*Aphrastura spinicauda*; Figure 1), a socially monogamous, cavity nesting furnariid distributed across a large latitudinal gradient in Chile and Argentina (30°S–55°S; Remsen, 2003). The Thorn‐tailed Rayadito is a resident species that breeds in natural cavities in temperate forests (Cornelius, 2008), but also occupies nestboxes in different habitats (Quilodrán et al., 2012; Tomasevic & Estades, 2006). Females lay one clutch of 3–5 eggs per breeding season during the Austral spring (Table 1). As described for other members of the family Furnariidae, both sexes contribute to incubation and feeding the young (Espíndola‐Hernández et al., 2017; Moreno et al., 2005, 2007). Genetic polyandry has been confirmed for rayaditos (Botero‐Delgadillo et al., 2019), but rates of EPP have not yet been quantified and described in this species. Furthermore, population differences in EPP rates may occur given marked environmental variation throughout the species’ extensive geographic range (see e.g., Ippi et al., 2011; Quirici et al., 2014). Using data from six breeding seasons and parentage analyses of a total of 266 broods, we estimate EPP rates for two populations located in the northern and southern parts of the species’ distribution. Additionally, we investigate the relationship between yearly variation in observed EPP rates and the social environment, using estimates of territory size of breeding pairs that were monitored as a proxy for population breeding density.

**Table 1:** Environmental, ecological and social characteristics of two populations of the Thorn‐tailed Rayadito breeding in Chile. Shown are means and standard deviations

	North (Fray Jorge)	South (Navarino)
Study period	2012–2017	2010–2015
Breeding season (typical)	Early September–late December	Early October–early January
Earliest record for a nesting attempt	26th August	21st September
Latest record for successful fledging	22nd December	20th January
Laying date^a^	27th September–29th October	10th October–26th November
Clutch size	3.0 ± 0.6 (*n* = 175 nests)	5.2 ± 0.5 (*n* = 124 nests)
Incubation period (days)	20.1 ± 2.2 (*n* = 175 nests)	17 ± 1.5 (*n* = 124 nests)
Brood size	2.7 ± 0.9 (*n* = 175 nests)	4.8 ± 1 (*n* = 124 nests)
Breeding territory diameter (m)	32.2 ± 7.6 (*n* = 189 pairs)	75.8 ± 13 (*n* = 131 pairs)
%Broods with EPO	20.7% (*n* = 140 broods)	14.3% (*n* = 126 broods)
%EPO	13.9% (*n* = 360 nestlings)	5.8% (*n* = 535 nestlings)

^a^ Recorded as the median date of the first egg for the first and last nesting attempt. The median value was calculated across years.

## 2 MATERIALS AND METHODS

### 2.1 Study populations

**Figure 2:**
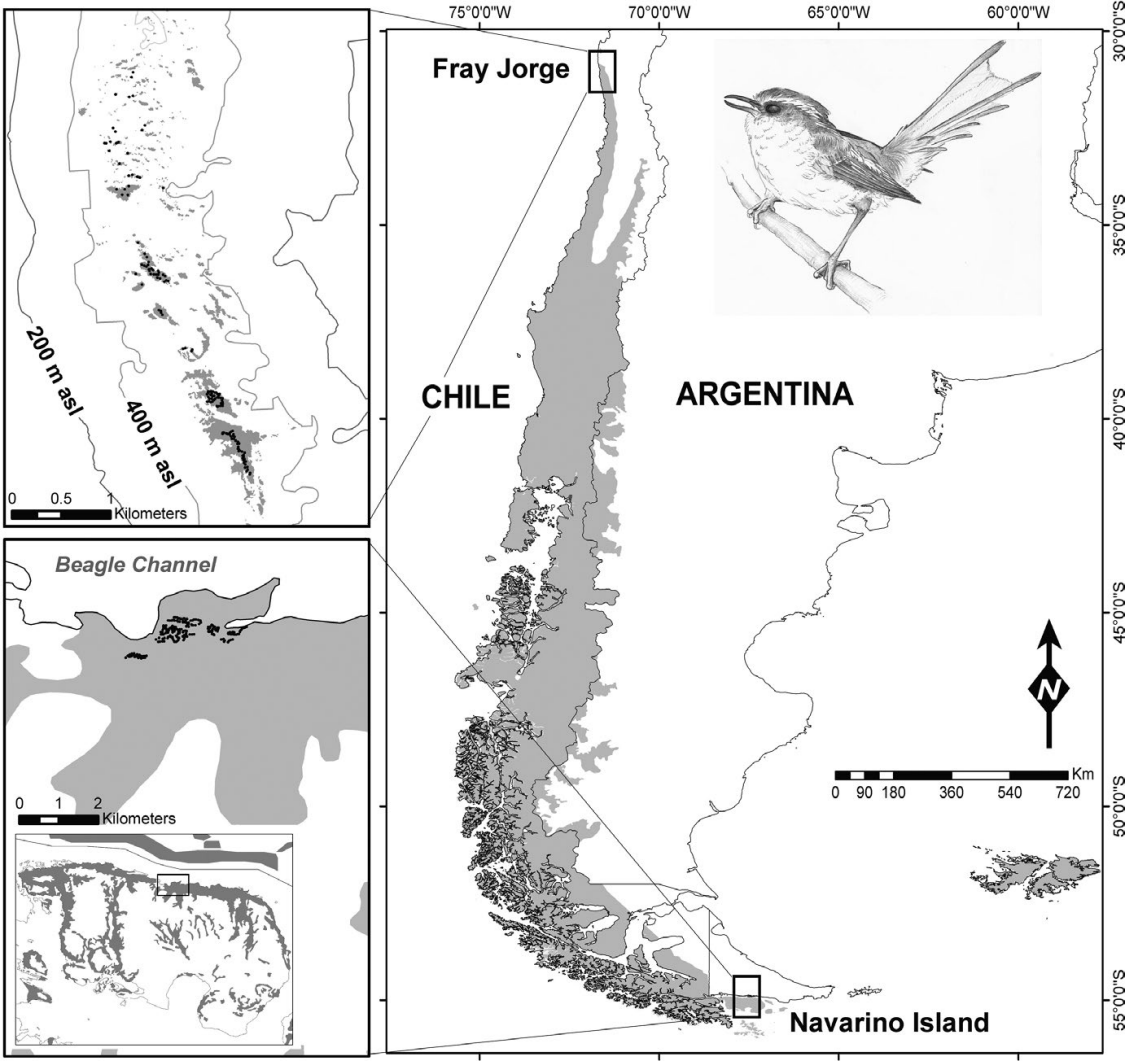
Breeding range of the Thorn‐tailed Rayadito and sampling localities in Fray Jorge National Park, north‐central Chile, and Navarino Island, southern Chile. Small panels show the distribution of nestboxes in forested areas (in gray) in each locality. Illustration of Thorn‐tailed Rayadito by Yifan Pei

As part of a long‐term study on the breeding biology of rayaditos, nestboxes were installed during 2006–2007 in two localities in north‐central and southern Chile (Figure 2). The northern locality is Fray Jorge National Park (30°38′S, 71°40′W), where the breeding season typically lasts four months, from early September to late December (Table 1). The southern locality—ca. 2,700 km south—is Navarino island (55°4′S, 67°40′W), where rayaditos usually breed during three months, between early October and early January (Table 1). A total of 101–157 and 171–222 nestboxes were provided in forest habitat in Fray Jorge and Navarino, respectively (Figure 2). At both sites, nestboxes were regularly distributed 20 to 25 m apart.

In the northern locality, rayaditos breed in a naturally fragmented relict of Valdivian temperate forest that is surrounded by a semiarid landscape (Figure 2), and that persists atop the coastal mountain range due to extensive water input from oceanic fog (del‐Val et al., 2006). This is considered a continental island population due to its isolation from other forested areas occupied by the species (Botero‐Delgadillo et al., 2020). Rayaditos from the southern population breed in extensive and rather continuous areas of sub‐Antarctic forest (Figure 2). Clutch and brood sizes are larger in the southern locality, while the incubation period is shorter (see e.g., Quirici et al., 2014; Table 1). The estimated breeding density is almost three times higher in the north than in the south (8.2 pairs/ha versus 2.8 pairs/ha, Botero‐Delgadillo, et al., 2017).

### 2.2 General procedures

Data were gathered during the breeding seasons of 2010–2017. A detailed description of field procedures is given in Botero‐Delgadillo, et al. (2017). Briefly, we checked nest boxes regularly between September and December during each year to record breeding phenology and measures of breeding productivity. We captured and marked all nestbox occupants (adults and nestlings) with numbered aluminum rings when nestlings were 12–14 days old. Adults breeding in natural cavities within the study sites were captured using mist nets. We obtained a blood sample (~15 µl) from all individuals by puncturing the brachial vein with a sterile needle. Blood samples were stored on FTA^™^ Classic Cards (Whatman^™^) for subsequent genetic analyses.

We extracted DNA from blood samples for genotyping and molecular sexing following the protocol described in Botero‐Delgadillo et al. (2017). We genotyped a total of 366 adults (183 from each population) and 895 nestlings from 266 broods at 12 autosomal, polymorphic microsatellite loci (for details see Botero‐Delgadillo et al., 2017). Adult sex was determined by amplifying the CHD locus using the primers P2/P8 (Griffiths et al., 1998).

### 2.3 Parentage analysis

We used multi‐locus genotypes for parentage analyses following the methods described in Botero‐Delgadillo et al. (2019). Analyses were performed in CERVUS version 3.0.7 (Kalinowski et al., 2007). Given the marker information content (the average number of alleles per locus was 10.4), and that maternity of the social female was confirmed for all nestlings (one mismatch with an offspring in eight cases), we determined whether the social male was also the genetic father with a combined probability of false exclusion <0.0001 in both populations. The social male was excluded when confidence around its assignment as the genetic father based on the logarithm‐of‐odds (LOD) score and the critical Delta value was <80%, or when it showed two or more mismatches with its putative offspring.

### 2.4 Estimation of territory size

Following the methods described in Valcu and Kempenaers (2008), Dirichlet tiles were used to estimate territory size for all breeding pairs in each population. We then calculated the mean territory diameter in each population for every breeding season. These values were used as a proxy for yearly breeding density. For these analyses, we considered all nesting attempts in nestboxes and in natural cavities recorded within the study sites. Dirichlet tesellation was performed with the package deldir (Turner, 2002) in R3.5.2 (R Core Team, 2018).

### 2.5 Frequency of EPO and population comparisons

We calculated the proportion of broods with at least one EPO and the total percentage of EPO in both populations (Table 1). Yearly values for these two parameters were also calculated (Table 2). Estimates of the frequency of extrapair paternity can be biased if not all eggs are genotyped, for example, due to hatching failure or brood reduction. Incomplete sampling occurred in 37 out of 189 clutches (19.6%) in the north and in 24 out of 126 clutches (19%) in the south. In total, we sampled 75% of all laid eggs (north: 68%, *n* = 529 eggs laid; south: 79%, *n* = 673 eggs).

**Table 2:** Yearly frequencies of extrapair paternity measured as the proportion of broods with at least one EPO and the total percentage of EPO in two populations of the Thorn‐tailed Rayadito breeding in Chile

Year	Percentage of broods with EPO		Percentage of EPO	
	North	South	North	South
2010	–	19.0 (*n* = 21)	–	7.7 (*n* = 91)
2011	–	15.0 (*n* = 20)	–	6.8 (*n* = 78)
2012	20.8 (*n* = 24)	0 (*n* = 14)	11.5 (*n* = 61)	0 (*n* = 61)
2013	16.7 (*n* = 24)	5.3 (*n* = 19)	10.5 (*n* = 53)	2.4 (*n* = 84)
2014	13.3 (*n* = 15)	18.8 (*n* = 32)	16.1 (*n* = 31)	9.6 (*n* = 137)
2015	25.1 (*n* = 24)	20.0 (*n* = 20)	16.9 (*n* = 59)	4.5 (*n* = 89)
2016	21.2 (*n* = 19)	–	8.2 (*n* = 49)	–
2017	23.5 (*n* = 34)	–	17.5 (*n* = 103)	–

We assessed whether the proportion of broods with EPO and the total percentage of EPO differed between populations using Fisher's exact tests. Because some breeding pairs were repeatedly sampled across years (48% and 30% of all adults were captured at least twice in the north and south, respectively), we applied the tests on a reduced dataset so as to minimize pseudo‐replication, only including the first breeding record for those birds with repeated data. We fitted a binomial mixed‐effects model to assess the effect of population (predictor variable) on the proportion of EPO (response) in nests with at least one EPO. Pair identity was included as random effect. This model was conducted in the lme4 package (Bates et al., 2015) in R.

We explored whether the frequency of EPP was linked to yearly variation in breeding density in each population, by calculating the Pearson's correlation coefficient between the mean territory diameter in each season and the yearly proportion of broods with EPO or the percentage of EPO.

## 3 RESULTS

Extrapair paternity was more common in the northern than in the southern population, both in terms of the proportion of broods with at least one EPO and in the total percentage of EPO (Table 1). However, only the latter was statistically supported, as indicated by Fisher's exact tests based on all data (266 broods and 895 nestlings; proportion of broods with EPO: *p* = .19; percentage EPO: *p* < .001) and the reduced dataset (179 broods and 658 nestlings; proportion of broods with EPO: *p* = .16; percentage EPO: *p* < .001).

The percentage of the brood that was EP in broods that contained at least one EPO ranged between 25% and 100% in the northern population (mean ± *SD*: 61.5% ± 29, *n* = 29), and between 14% and 50% in the southern population (mean ± *SD*: 28.7% ± 13, *n* = 18; mixed‐effects model with binomial error structure: *β_0_* ± *SD* (intercept) = −0.35 ± 0.38, *β* ± *SD* = −2.54 ± 1.09, *z* = −2.32, *p* = .02). Only in the northern population did we observe broods where the social male did not sire a single offspring (31% of 29 EP broods).

**Figure 3:**
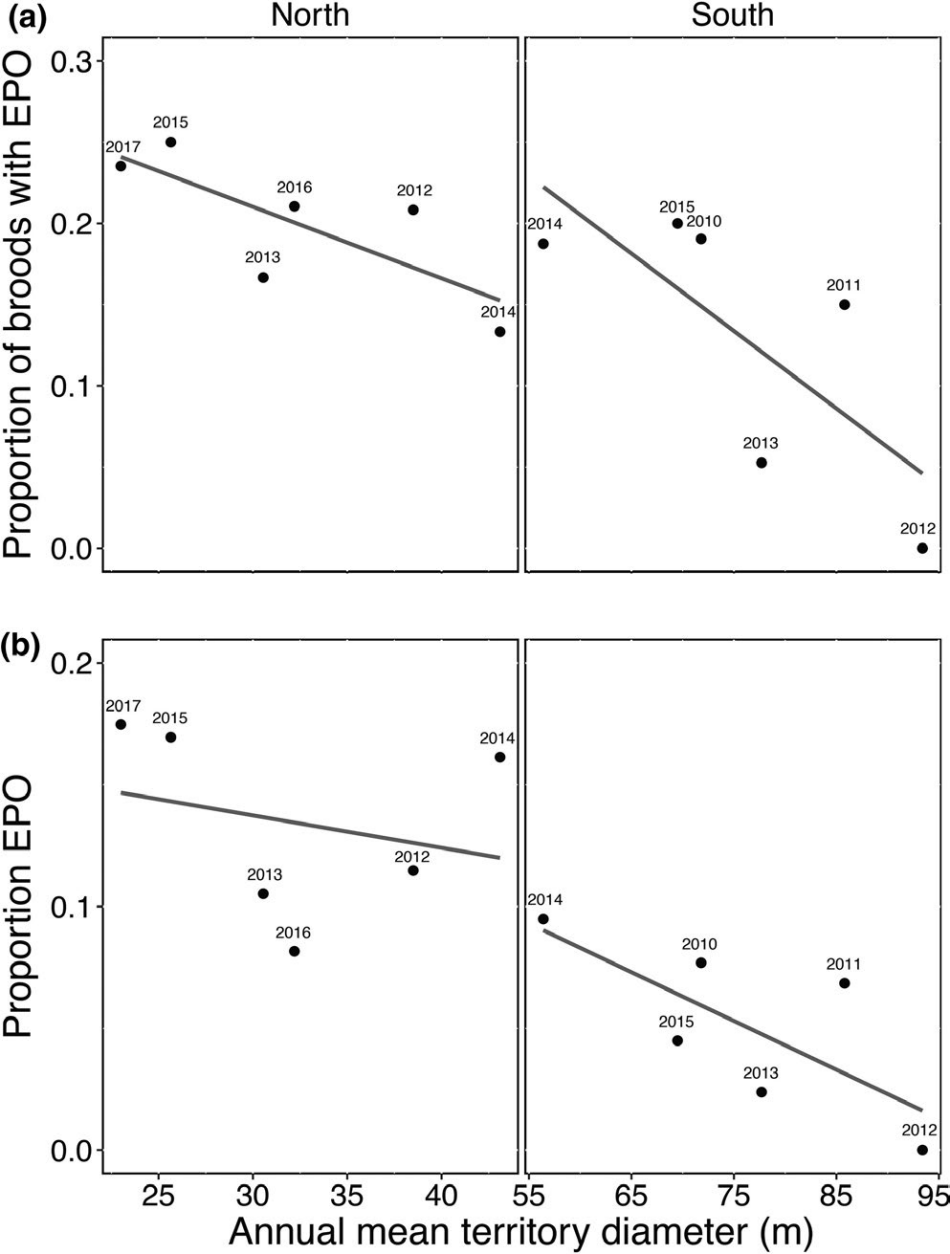
Relationship between the frequency of EPP and breeding density across years in two Chilean populations of the Thorn‐tailed Rayadito. (a) The proportion of broods with at least one EPO. (b) The percentage of extrapair offspring (EPO). Shown are annual values of mean breeding territory size as a proxy of variation in breeding density

The proportion of broods with EPO varied between 13% and 24% among years in the northern population and between 0% and 20% in the southern population (Table 2). Each year, 8%–18% and 0%–10% of all offspring were EPO, respectively (Table 2). Territories were generally larger in the southern population than in the north—reflecting a lower breeding density in the south—(Table 1), and we found a negative relationship between the frequency of EPP and the mean territory size across years (Figure 3). Correlations were moderate to high, but with weak statistical support due to small sample size (proportion of broods with EPO, north: *r* = −0.77, *p* = .07, south: *r* = −0.74, *p* = .08; percentage of EPO, north: *r* = −0.26, *p* = .62, south: *r* = −0.73, *p* = .09; *n* = 6 in all cases).

## 4 DISCUSSION

We found that the frequency of extrapair paternity in the Thorn‐tailed Rayadito (14%–21% broods with EPO; 6%–14% EPO) was similar to values reported for many other socially monogamous birds. Genetic polyandry has been detected in ~76% of the 255 biparental, socially monogamous species that have been studied to date, and the proportion of EPO is frequently < 20% (Brouwer & Griffith, 2019).

Information on EPP in Neotropical taxa is still scarce, particularly for the Suboscine passerines (see Brouwer & Griffith, 2019; Griffith et al., 2002). Our results are among the first to quantify EPP rates in a species of the furnarioid clade (i.e., the tracheophone suboscines, with ~700 species; see Chesser, 2004; Moyle et al., 2009; Remsen et al., 2020). A first study of 89 offspring from 50 broods of the Black‐crowned Antshrike (*Thamnophilus atrinucha*) found that 4% of broods contained EPO and that 3.4% of all offspring were EPO (Tarwater et al., 2013). A second study of 120 offspring from 46 broods of Rufous Hornero (*Furnarius rufus*) reported values of 6.5% and 3.3%, respectively (Diniz et al., 2019). Long‐term pair bonds and extensive biparental care, typically found in thamnophilids and furnariids (see Remsen, 2003; Skutch, 1996; Zimmer & Isler, 2003), are usually invoked as explanations for these low values of EPP, as this could lead to potentially high costs of retaliation by males (Tarwater et al., 2013).

We collected data from two populations that are likely subjected to contrasting selective pressures (Botero‐Delgadillo, et al., 2017; Quirici et al., 2014). The EPP rates observed in the northern population were markedly higher than those in the southern population and those reported for other furnarioid taxa (Diniz et al., 2019; Tarwater et al., 2013). Overall, mating strategies in the Furnarioidea remain poorly studied, and evidence for intraspecific variation in EPP is scarce. Further study on mating systems and breeding behavior is thus required to understand the factors potentially driving intraspecific differences in this ecologically diverse group.

Previous studies suggest that neither latitude, nor life‐history offer a general explanation for intraspecific variation in EPP in socially monogamous birds (Brouwer & Griffith, 2019). However, Brouwer and Griffith (2019) found a negative association between the frequency of EPP and latitude for noncolonial species from the northern hemisphere for which EPP rates were reported for at least 10 different populations (e.g., Blue Tit, Great Tit, Pied Flycatcher *Ficedula hypoleuca*; see also Møller & Ninni, 1998). These results support the idea of a trade‐off between searching for extrapair matings—which may be less costly at higher latitudes because of higher breeding synchrony—and parental investment—which is expected to be higher at higher latitudes due to shorter breeding seasons (see below).

Although we only compared two populations, our data conform to the pattern documented in other socially monogamous species in the northern hemisphere. The frequency of EPP was lower in the southern, more seasonal environment. Testing the underlying reasons behind this difference is beyond the scope of this study, but we briefly discuss some nonmutually exclusive potential explanations. First, the shorter breeding season in the southern locality imposes time constraints on rayaditos (Botero‐Delgadillo et al., 2017), which could generate a trade‐off between the benefits of extrapair matings that are facilitated by a higher breeding synchrony, and the costs associated with the loss of paternal care and the need to match a breeding attempt with a brief period of high resource availability. For instance, the need for male parental care in emberizid sparrows is likely stronger in high‐latitude and high‐elevation sites, and consequently EPP rates might be lower (Bonier et al., 2014).

Second, the higher breeding density in the northern locality—possibly a result of the reduced availability of breeding habitat due to forest fragmentation (Gutiérrez et al., 2008)—could facilitate encounters between potential extrapair mates (Westneat et al., 1990), thus increasing EPP rates. In support of this idea, we also found a negative relationship between annual mean territory size—a proxy of breeding density—and both the percentage of broods with EPO and the proportion of EPO, suggesting that density could partially explain the observed variation in EPP between and within populations. The statistical evidence for this relationship is weak, presumably because of a lack of statistical power. Whether variation in EPP rates (and in breeding density) are linked to other environmental variables such as variation in temperature or precipitation regimes needs further evaluation. Variation in rainfall could affect resource availability, which might in turn affect spatio‐temporal variation in breeding density and EPP.

Several other factors need to be evaluated to better understand between‐ and within‐population variation in EPP in the Thorn‐tailed Rayadito. These include differences in breeding synchrony (Westneat et al., 1990), variation in food availability (Westneat & Mays, 2005), and annual variation in demographic parameters. It might also be useful to sample additional populations to improve our understanding of the potential effect of latitude and how it relates to environmental—for example, temperature or precipitation regimes—or life‐history variation.

The current lack of information on the breeding biology of tropical and southern hemisphere species hinders our understanding of variation in avian life histories (see Xiao et al., 2017). More reports on EPP rates are necessary to determine the patterns of taxonomic and geographic variation in mating strategies in Neotropical birds, and to better understand the differences in ecological dynamics between northern and southern hemisphere populations.

## CONFLICT OF INTEREST

None declared.

## AUTHOR CONTRIBUTION

**Esteban Botero‐Delgadillo:** Conceptualization (lead); Data curation (lead); Formal analysis (lead); Funding acquisition (supporting); Investigation (equal); Methodology (equal); Resources (equal); Visualization (lead); Writing‐original draft (lead). **Verónica Quirici:** Funding acquisition (supporting); Investigation (equal); Methodology (equal); Resources (equal). **Yanina Poblete:** Funding acquisition (supporting); Investigation (equal); Methodology (equal); Resources (equal). **Silvina Ippi:** Funding acquisition (supporting); Investigation (equal); Resources (equal). **Bart Kempenaers:** Funding acquisition (supporting); Investigation (equal); Resources (equal); Supervision (equal); Writing‐review & editing (lead). **Rodrigo Vasquez:** Funding acquisition (lead); Investigation (equal); Project administration (lead); Resources (equal); Supervision (equal); Writing‐review & editing (supporting).

## Data Availability

Genotype data and the dataset analyzed during the current study are available from the Dryad Digital Repository (https://doi.org/10.5061/dryad.hdr7sqvg3).
